# Ultra-accurate sequencing reveals an extreme transmission bottleneck in a deep-sea clam symbiosis

**DOI:** 10.64898/2026.06.29.735038

**Published:** 2026-07-02

**Authors:** Cade Mirchandani, Evan Pepper-Tunick, Landen Gozashti, Shelbi Russell, Russ Corbett-Detig

**Affiliations:** 1 Department of Biomolecular Engineering, University of California Santa Cruz, Santa Cruz, CA, United States; 2 Genomics Institute, University of California Santa Cruz, Santa Cruz, CA, United States; 3 Institute for Systems Biology, Seattle, Washington, USA; 4 Molecular Engineering and Sciences Institute, University of Washington, Seattle, Washington, USA; 5 Department of Integrative Biology, University of California, Berkeley; 6 Buck Institute for Research On Aging, Novato, CA, USA

## Abstract

Vertically transmitted symbionts experience progressive genome degradation driven by transmission bottlenecks each host generation that reduce genetic diversity and promote fixation of deleterious mutations. Direct estimates remain rare because inference requires scarce parent–offspring samples and sequencing sensitive enough to detect rare variants. Here, we investigate symbiont transmission bottlenecks in a vesicomyid clam by deeply sampling within-host endosymbiont genetic diversity using two ultra-accurate sequencing methods. Demographic modeling revealed an effective bottleneck size of approximately eight symbionts (95% CI: 1–17 genomes) per host generation. This estimate is sharply reduced relative to prior cytological estimates of bottleneck census size, with important implications for understanding the rate and dynamics of endosymbiont genome degradation.

## Introduction

Obligate endosymbionts experience progressive genome reduction driven by small effective population sizes, reduced recombination, and recurrent transmission bottlenecks that intensify genetic drift (1). Over time, this process drives the irreversible accumulation of deleterious mutations through Muller’s ratchet (2). The severity of genome erosion depends critically on the number of genetically distinct symbiont lineages transmitted from parent to offspring, termed the transmission bottleneck size (3).

Despite its importance in determining evolutionary outcomes, the transmission genetic bottleneck size has proven difficult to measure directly. Existing estimates typically rely on cell counts rather than genetically distinct lineages, and these may differ substantially: cell counts may not reflect genetically distinct founders and the tissue sampled may not capture the maximal bottleneck. In the vesicomyid Calyptogena okutanii, roughly 400 symbiont cells are transmitted per egg, each containing approximately 10 genome copies (4), but because the transmission bottleneck may precede embryo provisioning, these cytological counts may not reflect the number of genetically distinct founders. In the aphid-Buchnera system, approximately 1,800 symbiont cells are transmitted per egg (5), yet intergenerational symbiont allele frequency dynamics suggest within-host effective population sizes of only 10–20 genomes. Obtaining parent–offspring samples is impractical in many systems including deep-sea clams, and standard sequencing lacks the sensitivity to characterize within-host allele frequency variation.

Although strict vertical transmission is rare among marine symbioses (6), the vesicomyid *Calyptogena magnifica* and its sulfur-oxidizing symbiont Candidatus *Ruthia magnifica* exhibit predominantly vertical transmission (7), which has likely dominated for much of their estimated 70–80 million year obligate association, making this system particularly well-suited for studying endosymbiont bottleneck dynamics. Despite this ancient association, the symbiont genome (~1.2 Mb) remains larger and less degraded than terrestrial insect symbiont counterparts of comparable age, possibly due to occasional horizontal transmission and recombination (8), but the magnitude of the transmission bottleneck and its implications for genome degradation remain unknown. Here, we use two ultra-accurate sequencing methods to detect rare genetic variants within a single host individual and infer the effective transmission bottleneck size through demographic modeling.

## Results and Discussion

**Figure 1.**
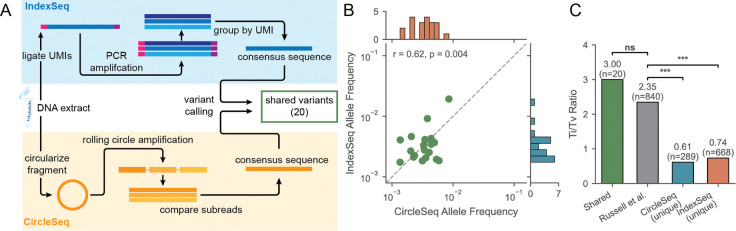
Variant validation. (A) Overview of sequencing approaches and variant calling pipeline. (B) Scatter plot showing allele frequency correlation between CircleSeq and IndexSeq at 20 shared variant positions, with marginal frequency distributions for each method. (C) Transition-to-transversion (Ti/Tv) ratios for shared variants, Russell et al. (2020) variants, and variants unique to each sequencing method. Chi-squared tests compare each category to Russell et al.; shared variants show no significant difference (χ^2^ = 0.05, p = 0.82), while variants unique to CircleSeq (χ^2^ = 92.35, p < 0.001) and IndexSeq (χ^2^ = 116.39, p < 0.001) differ significantly.

We applied two ultra-accurate sequencing methods to the same DNA extraction from the gill tissue of a single vesicomyid clam collected from the Galápagos Rift (0°48.2’N, 86°13.9’W, 2,461 m depth). Whole-genome alignment of the symbiont consensus assembly to the *Ca.* Ruthia magnifica reference genome (GCF_000015105.1) yielded 99.98% nucleotide identity (212 SNPs and 24 indels across 1.16 Mb) and 100% identity at the 16S rRNA gene. Because *Ca.* Ruthia magnifica is an obligate, species-specific symbiont of *C. magnifica (9, 10)*, we infer the host accordingly. From this sample, circle sequencing (11, 12) identified 289 variants while index sequencing (13) identified 668 variants (Fig 1A). In both datasets, variants were rare (Fig 1B), with allele frequencies consistent with a largely clonal endosymbiont population.

The significant overlap in variant calls and plausible biological properties of variants detected by each method indicates both methods identify a true biological signal. The two approaches detected largely distinct variant sets, with 20 variants shared between them, presumably due to extremely low allele frequencies and method-specific systematic errors. This overlap was significantly greater than expected by chance (permutation test, p < 0.001). Allele frequencies at shared positions were significantly positively correlated between methods (Pearson r = 0.62, p = 0.004) and ranged from 0.0014 to 0.0193 (Fig 1B), which suggests that for these overlapping variants, both methods detected a largely concordant biological signal. Finally, the transition-to-transversion (Ti/Tv) ratio of shared variants was comparable to that of polymorphic variants across a population of *C. Ruthia magnifica* sequenced with short-reads (Russell et al. 2020, p = 0.82, Fig 1C), whereas variants private to either sequencing method demonstrated substantially reduced Ti/Tv ratios (Fig 1C). Together, these results suggest that the shared variants represent genuine biological mutations suitable for demographic inference.

**Figure 2.**
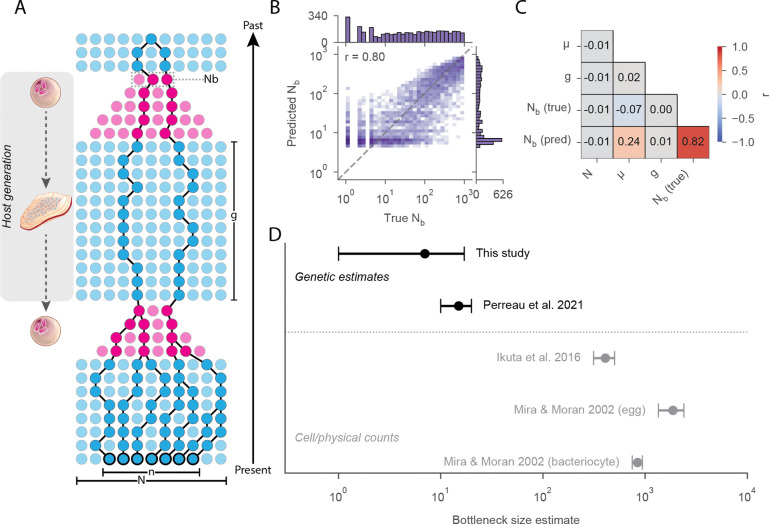
Bottleneck inference. (A) Two-phase demographic model. A host generation is shown on the left, with egg and adult clam cartoons indicating transmission. Circles represent symbiont lineages across generations; blue and pink denote stasis and bottleneck phases. Bold-outlined circles indicate sampled lineages (n). N, effective population size after host development; Nb, transmission bottleneck size; g, generations after initial expansion; per-site mutation rate (μ) not depicted. (B) Random forest performance on held-out simulations, with marginal distributions of true and predicted Nb. (C) Pairwise correlations among nuisance parameters (N, μ, g) and true/predicted Nb. (D) Bottleneck estimates across symbiont systems: genetic (black, effective genomes) vs. census (gray, microscopy).

To infer the transmission bottleneck size from these shared variants, we performed demographic modeling of endosymbiont populations using a type of approximate Bayesian computation. We developed a discrete generation backward-in-time coalescent simulator to model symbiont genealogies under a two-phase demographic model of symbiont transmission (Fig 2A), and trained a random forest model on the resulting simulated allele frequency spectra to predict the bottleneck population size. The model predicted log_10_(Nb) with high accuracy (r = 0.80 on held-out simulations; Fig 2B), and predictions were largely independent of nuisance parameters population size (N), per-site mutation rate (μ), and stasis generations (g), though Nb weak residual correlation with μ (r = 0.24) because both parameters influence the number of detectable variants (Fig 2C). Applied to our 20 high confidence variants, the model estimated an effective bottleneck estimate of approximately 8 symbionts (95% CI: 1–17) per host generation (Fig 2D).

Our estimated population bottleneck size is comparable to effective population size estimates in terrestrial insect endosymbionts, and substantially smaller than those derived from microscopy. Experimental evolution in aphid–Buchnera estimated within-host effective population sizes of only 10–20 (14), despite vastly different transmission mechanisms: vesicomyid symbionts are attached extracellularly to broadcast-spawned eggs (4), whereas Buchnera is transferred intracellularly from maternal bacteriocytes to developing embryos. Even under this severely restricted bottleneck, the *C. ruthia magnifica* genome remains substantially larger than terrestrial insect symbiont counterparts of comparable evolutionary age. This contrast lends further support to the inference that occasional horizontal transmission and recombination are more common in this clam population and act to maintain genome integrity in this system, counteracting the degenerative effects of drift even under extreme bottlenecks (8).

Genetic estimates of transmission bottlenecks complement existing census-based observations by indirectly inferring the number of genetically independent lineages transmitted each host generation. For example, in the related *C. okutanii*, approximately 400 symbiont cells are found on host egg surfaces (4), and in aphid–*Buchnera*, roughly 1,800 cells are transmitted per egg (5). Our estimate is orders of magnitude below these census counts, consistent with the expectation that effective and census population sizes diverge substantially in microbial systems (15). While census counts provide valuable insight into the physiology and ecology of symbiont transmission, the forces shaping genome erosion tend to be driven by effective population size. Therefore, genetic estimates across diverse host phylogenies, transmission mechanisms, and reproductive modes will be necessary for accurately parameterizing the evolutionary forces shaping endosymbiont genome trajectories.

## Methods

We called variants from two ultra-accurate sequencing methods applied to a single DNA extraction from *C. magnifica* gill tissue. Bottleneck size was inferred by training a random forest regressor on simulated allele frequency spectra generated under a discrete backward-in-time demographic model. Full details are in SI Appendix.

## Supplementary Material

Supplement 1

## References

[1] Wernegreen J. J., Genome evolution in bacterial endosymbionts of insects. Nat. Rev. Genet. 3, 850–861 (2002). 12415315 10.1038/nrg931

[2] Pettersson M. E., Berg O. G., Muller’s ratchet in symbiont populations. Genetica 130, 199–211 (2007). 16924405 10.1007/s10709-006-9007-7

[3] Kaltenpoth M., Goettler W., Koehler S., Strohm E., Life cycle and population dynamics of a protective insect symbiont reveal severe bottlenecks during vertical transmission. Evol. Ecol. 24, 463–477 (2010).

[4] Ikuta T., , Surfing the vegetal pole in a small population: extracellular vertical transmission of an “intracellular” deep-sea clam symbiont. R Soc Open Sci 3, 160130 (2016). 27293794 10.1098/rsos.160130 PMC4892456

[5] Mira A., Moran N. A., Estimating population size and transmission bottlenecks in maternally transmitted endosymbiotic bacteria. Microb. Ecol. 44, 137–143 (2002). 12087426 10.1007/s00248-002-0012-9

[6] Russell S. L., Transmission mode is associated with environment type and taxa across bacteria-eukaryote symbioses: a systematic review and meta-analysis. FEMS Microbiol. Lett. 366 (2019). 10.1093/femsle/fnz013 30649338

[7] Cary S. C., Giovannoni S. J., Transovarial inheritance of endosymbiotic bacteria in clams inhabiting deep-sea hydrothermal vents and cold seeps. Proc. Natl. Acad. Sci. U. S. A. 90, 5695–5699 (1993). 8100068 10.1073/pnas.90.12.5695 PMC46788

[8] Russell S. L., , Horizontal transmission and recombination maintain forever young bacterial symbiont genomes. PLoS Genet. 16, e1008935 (2020). 32841233 10.1371/journal.pgen.1008935 PMC7473567

[9] Newton I. L. G., , The Calyptogena magnifica chemoautotrophic symbiont genome. Science 315, 998–1000 (2007). 17303757 10.1126/science.1138438

[10] Stewart F. J., Young C. R., Cavanaugh C. M., Lateral symbiont acquisition in a maternally transmitted chemosynthetic clam endosymbiosis. Mol. Biol. Evol. 25, 673–687 (2008). 18192696 10.1093/molbev/msn010

[11] Penunuri G. A., Pepper-Tunick E. A., McBroome J., Corbett-Detig R., Russell S., EMS mutation and SNP detection in intracellular Wolbachia genomes. bioRxiv (2026). 10.1128/msystems.00660-26 PMC13483387 42405770

[12] Lou D. I., , High-throughput DNA sequencing errors are reduced by orders of magnitude using circle sequencing. Proc. Natl. Acad. Sci. U. S. A. 110, 19872–19877 (2013). 24243955 10.1073/pnas.1319590110 PMC3856802

[13] Kennedy S. R., , Detecting ultralow-frequency mutations by Duplex Sequencing. Nat. Protoc. 9, 2586–2606 (2014). 25299156 10.1038/nprot.2014.170 PMC4271547

[14] Perreau J., Zhang B., Maeda G. P., Kirkpatrick M., Moran N. A., Strong within-host selection in a maternally inherited obligate symbiont: Buchnera and aphids. Proc. Natl. Acad. Sci. U. S. A. 118 (2021). 10.1073/pnas.2102467118 PMC8536349 34429360

[15] Bobay L.-M., Ochman H., Factors driving effective population size and pan-genome evolution in bacteria. BMC Evol. Biol. 18, 153 (2018). 30314447 10.1186/s12862-018-1272-4 PMC6186134

