# Supplementary material for: Ultra-accurate sequencing reveals an extreme transmission bottleneck in a deep-sea clam symbiosis
Source: bioRxiv. 2026 Jul 2:2026.06.29.735038. Preprint. [Version 1] doi: 10.64898/2026.06.29.735038 (PMC13345212; doi:10.64898/2026.06.29.735038)
Supplement: Supplement 1 [file NIHPP2026.06.29.735038v1-supplement-1.pdf]

bioRxiv preprint doi: https://doi.org/10.64898/2026.06.29.735038; this version posted July 3, 2026. The copyright holder for this preprint (which was not certified by peer review) is the author/funder, who has granted bioRxiv a license to display the preprint in perpetuity. It is made available under a CC-BY 4.0 International license.

SI Appendix

Sample Collection

Gill tissue was collected from a vesicomyid clam at the Galápagos Rift hydrothermal vent field (0°48.2'N, 86°13.9'W, ~2,461 m depth) on May 29, 1990 (Dive 2224) and stored at −80°C. The specimen was field-identified as a vesicomyid. To confirm species identity, we aligned a Pilon-polished (Walker et al. 2014) symbiont consensus assembly to the *Ca.* Ruthia magnifica reference genome (GCF_000015105.1, ASM1510v1) using minimap2 (Li 2018) with the asm5 preset. The alignment recovered 99.98% nucleotide identity across the full 1.16 Mb reference genome (212 SNPs, 10 insertions totaling 20 bp, and 14 deletions totaling 38 bp). We additionally extracted the 16S rRNA gene (reference positions 1,081,269–1,082,814) from both the reference and our assembly and compared them using BLASTn (Altschul et al. 1990), yielding 100% identity over 1,546 bp. These values fall within expected intraspecific variation and confirm the symbiont as *Ca.* Ruthia magnifica, consistent with the host being *Calyptogena magnifica*. Tissue was sterile-dissected or subsectioned from previously sterile-dissected gill tissue as described in Russell et al. (2020). DNA was extracted from gill tissue of a single individual for use in both sequencing approaches.

Sequencing and Variant Calling

We used two complementary approaches to detect low-frequency variants from the same DNA extraction. For circle sequencing, DNA fragments are circularized and amplified via rolling circle amplification, generating tandem copies of each original molecule; we aligned these reads to the reference genome using BWA-MEM, realigned subsequences using Smith-Waterman alignment, and collapsed them into consensus sequences, masking any base with disagreement across subreads (Lou et al. 2013). For index sequencing, molecular barcodes are ligated to DNA fragments, allowing reads derived from the same original molecule to be identified after sequencing. Reads sharing a barcode are grouped into families and collapsed into single-strand consensus sequences (SSCS), in which each base is determined by majority agreement among family members. While the Duplex-Seq-Pipeline (Kennedy et al. 2014) can further combine complementary SSCS from opposite strands into duplex consensus sequences (DCS), duplex formation requires sufficient sequencing depth to recover both strands of each original molecule. Our target genome yielded insufficient strand pairing for reliable DCS generation, so we used SSCS for variant calling, which retains higher yield at a moderately higher error rate ($\sim 10^{-4}$) compared to DCS ($\sim 10^{-6}$; Lou et al. 2013).

We generated pileups from consensus sequences using samtools mpileup (Li et al. 2009) and called variants using a custom Python script (https://github.com/cademirch/symbiont_transmission). We applied identical filters to both methods. First, to identify error-prone genomic regions, we modeled the expected number of variants per 1 kb window as a Poisson distribution with rate $\lambda = V \times W / G$, where V is the total

bioRxiv preprint doi: https://doi.org/10.64898/2026.06.29.735038; this version posted July 3, 2026. The copyright holder for this preprint (which was not certified by peer review) is the author/funder, who has granted bioRxiv a license to display the preprint in perpetuity. It is made available under a CC-BY 4.0 International license.

variant count, W is the window size, and G is the genome size. We excluded windows in which the observed variant count exceeded the 99th percentile of this expectation, removing regions with anomalously high variant density indicative of systematic alignment or sequencing artifacts. We computed outlier windows independently for each method and excluded the union of both sets. Second, we required a minimum of 3 supporting consensus reads and a minimum allele frequency of 0.15%, approximately one order of magnitude above the per-base error rates for circle sequencing (~$2–5 \times 10^{-5}$) and single-strand consensus sequencing (~10^{-4}; Lou et al. 2013). We calculated allele frequencies as alternate read counts divided by total depth. We defined shared variants as positions with the same alternate allele called independently by both sequencing methods. At matched consensus depths, the two methods produced comparable variant counts, and the higher number of method-specific variants in circle sequencing is consistent with its broader depth distribution (336–6,012×) compared to index sequencing (1,269–1,871×).

Backward Time Symbiont Population Simulation

We developed a discrete-generations backward-in-time population simulator to model symbiont genealogies under an alternating-phase demographic model (https://github.com/cademirch/symbiont_transmission). The simulation tracks a sample of n lineages backward through time. The demographic model cycles through successive host generations, each consisting of two phases: a stasis phase where the symbiont population maintains effective size N for g generations, and a growth phase modeling post-transmission population expansion. In forward time, N_b symbionts colonize a new host and expand via binary fission to N over $\log_2(N/N_b)$ generations. In backward time, we model this as an exponential contraction where the effective population size halves each generation from N to N_b, increasing the coalescent rate as lineages approach the transmission event. In each generation, we draw the expected number of coalescent events from a Poisson distribution with rate $k(k-1)/2N$, where k is the current number of active lineages. For each coalescent event, we choose two lineages uniformly at random, merge them into a single ancestral lineage, and record the branch lengths from each child to the new parent. Note that we explicitly do not allow more than two lineages to share a single common ancestor in one generation – i.e., consistent with binary cell division. So long as $n << N$, merges including three or more lineages are exceedingly rare. The simulation alternates between stasis and bottleneck phases, proceeding backward in time until all sampled lineages coalesce to a single common ancestor.

We place mutations on the resulting genealogy following a Poisson process. We aggregate branch lengths by the number of descendant samples: a mutation occurring on a branch with d descendants appears at frequency d/n in the sample. For each descendant class, we compute the expected number of mutations as the product of the total branch length in that class and the genome-wide mutation rate (per-site rate × genome size) μ, and draw the realized count from a Poisson distribution with this expectation. To model the observation process, we independently subsample each mutation through both sequencing methods. For each mutation at true sample frequency $p = d/n$, we draw a consensus depth D from the empirical depth distribution of each sequencing method and then draw the observed alternate read count from Binomial(D, p). While

bioRxiv preprint doi: https://doi.org/10.64898/2026.06.29.735038; this version posted July 3, 2026. The copyright holder for this preprint (which was not certified by peer review) is the author/funder, who has granted bioRxiv a license to display the preprint in perpetuity. It is made available under a CC-BY 4.0 International license.

the hypergeometric distribution more exactly models sequencing as sampling without replacement, the probability of sampling an alternate read is sufficiently low that replacement has negligible effect on subsequent draws, and the Binomial provides an equivalent approximation. We consider a mutation detected by a given method if the alternate read count meets or exceeds the minimum threshold. This framework captures the joint effects of demographic history, mutational input, and the stochastic detection process of each sequencing method, allowing us to evaluate how well observed variant counts and allele frequency spectra match expectations under different demographic scenarios.

Bottleneck Size Estimation

To estimate the transmission bottleneck size, we trained a random forest regressor on simulated allele frequency spectra generated under varying demographic scenarios. We sampled 50,000 parameter combinations using a Sobol quasi-random sequence to achieve uniform coverage of the four-dimensional parameter space: bottleneck size (N_β) from 1 to 1,000 (log-uniform), effective population size (N) from 10^8 to 10^{10} (log-uniform), per-site mutation rate (μ) from 10^{-10} to 10^{-8} per generation (log-uniform), and stasis generations (g) from 100 to 10,000 (uniform). For each parameter combination, we generated an observable allele frequency spectrum using the sequencing observation model described above, applying the same variant-calling thresholds used on empirical data and retaining only mutations detected by both methods. We binned allele frequencies into 10 log-spaced bins spanning 1.5×10^{-3} to 1, with the lower bound set to match our minimum detectable allele frequency of 0.15%. Bins were computed independently for each sequencing method, yielding a 20-dimensional feature vector per simulation.

We trained a random forest regressor (scikit-learn; (Pedregosa et al. 2012)) to predict $\log_{10}(N_\beta)$ from these feature vectors, holding out 20% of samples as a test set. We selected hyperparameters via randomized search over 100 iterations of 5-fold cross-validation, minimizing mean squared error, yielding a forest of 492 trees with maximum depth 10, minimum samples per split of 9, and 30% of features considered at each split. We evaluated model performance on the held-out test set by R^2, root mean squared error (RMSE), and mean absolute error (MAE), and confirmed generalization with 5-fold cross-validated R^2 on the training set. We applied the trained model to our empirical shared variants and estimated prediction uncertainty from the distribution of predictions across individual trees in the forest, reporting the mean as the point estimate and the 2.5th and 97.5th percentiles as a 95% confidence interval.
